# The experience of receiving a letter from a cancer genetics clinic about risk for hereditary cancer

**DOI:** 10.1038/s41431-024-01551-9

**Published:** 2024-02-14

**Authors:** Anna Öfverholm, Per Karlsson, Anna Rosén

**Affiliations:** 1 Institute of Clinical Sciences, Department of Oncology, Sahlgrenska Academy, Gothenburg University, Göteborg, Sweden; 2 Department of Diagnostics and Intervention, Oncology, Umeå University, Umeå, Sweden

**Keywords:** Genetic counselling, Genetic testing

## Abstract

Direct contact may be an option for supporting disclosure in families with hereditary cancer risk. In this qualitative interview study, we explored how healthy at-risk relatives experience receiving a letter with information about hereditary cancer directly from healthcare rather than via a relative. The study is part of an ongoing multicentre randomised clinical trial in Sweden that evaluates the effectiveness of direct letters from cancer genetics clinics to at-risk relatives. After conducting semi-structured interviews with 14 relatives who had received a letter and contacted the clinic, we analysed the data using thematic analysis. The relatives had different levels of prior knowledge about the hereditary cancer assessment. Many had been notified by family that a letter was coming but some had not. Overall, these participants believed healthcare-mediated disclosure could complement family-mediated disclosure. They expressed that the letter and the message raised concerns and a need for counselling, and they wanted healthcare to be accessible and informed when making contact. The participants found the message easier to cope with when they had been notified by a family member beforehand, with a general attitude that notifying relatives was the appropriate step to take. They thought healthcare should help patients with the disclosure process but also guard the right of at-risk relatives to be informed. The findings support a direct approach from healthcare as a possible complement to an established model of family-mediated risk disclosure, but implementation must be made within existing frameworks of good practice for genetic counselling.

## Introduction

The results from genetic testing for hereditary cancer risk can have implications not only for treatment of the patient but also with regard to risk assessment for relatives. Identifying high risk for hereditary cancer such as breast and ovarian cancer syndrome (HBOC) or Lynch syndrome is important. Awareness of risk supports timely access to targeted programs for early detection and prevention, including risk-reducing surgery, that decrease cancer incidence and mortality [1, 2]. When healthy at-risk relatives are identified as carriers of a familial variant in a cancer-predisposing gene, they can be offered such prevention programs. Individuals in families with a high occurrence of breast cancer or colorectal cancer but negative genetic screening also can be offered prevention programs for early detection [3–6].

Clinical practice in Sweden and elsewhere in Europe, in the United States, and in Australia has adopted a family-mediated disclosure model. In this model, the genetic healthcare professional is responsible for counselling the patients about the importance of informing their relatives about the genetic risk. This ethically and legally well-established model can be debated when clinical practices are changing: The indications for genetic testing are extending, and there is a steady increase in patients and families who need to be counselled not only at cancer genetics clinics but also in the context of mainstreamed testing routines. Mainstreamed testing occurs when non-genetic healthcare professionals, such as oncologists and surgeons, offer genetic testing as a part of ongoing cancer treatment [7]. This kind of testing is a topic of discussion in Sweden and has been partially implemented in some healthcare regions. As more individuals learn about positive genetic testing results in different healthcare and counselling settings, it is reasonable to develop existing counselling models to support disclosure in the family.

We also argue that healthcare has a moral responsibility to ensure that information is made available to at-risk relatives, but also that healthcare professionals do not have a duty to ensure that patients take moral responsibility for the health of others. This is an argument for developing approaches for direct contact [8].

One recent meta-analysis of compiled data on current established practices in family-mediated disclosure showed that about 70% of at-risk relatives are informed about hereditary risk. Of these, about 43% undergo genetic testing for the familial variant [9]. Another meta-analysis on hereditary cancer risk disclosure showed that with family-mediated disclosure, the uptake of genetic counselling in relatives is about 35%, whereas the uptake is almost doubled (63%) with a direct contact from healthcare to relatives [10]. In contrast, an observational study of the impact of new guidelines for cascade screening including direct contact in the Netherlands did not show such an effect [11].

The attitudes and reactions of at-risk relatives to direct contact and potentially unsolicited disclosure by healthcare are not well known. A handful of studies with data from both questionnaires and interview studies show that at-risk relatives seem to perceive it as an acceptable but complicated addition to current practices [11–15].

We conducted this qualitative interview study in the context of an ongoing Swedish multi-centre randomised clinical trial [16] evaluating the offer of direct letters from healthcare to at-risk relatives regarding hereditary cancer risk and the rate of relatives contacting a cancer genetics clinic. For this investigation, we explored the experiences and attitudes of at-risk relatives who received a direct letter.

## Methods

### Study design and data collection and analysis

We performed a qualitative interview study in which participants were at-risk relatives who had received a letter from a cancer genetics clinic. The letter was unsolicited and the relatives’ first contact with the clinic. It was sent by registered mail (the relative received a notification and retrieved the letter after identifying themselves at a postal delivery unit) after patients’ consent as an intervention within a Swedish clinical randomised controlled trial that compared direct contact with standard care [16]. The letter was adjusted to the specific familial risk, had a familial serial number, disclosed hereditary risk information, and offered genetic counselling and testing (for an example of the letter, see supplementary information).

If relatives contacted the cancer genetics clinic, a genetic counsellor and research nurse invited them to participate in the study, and we scheduled an interview within 2 weeks of the contact. The inclusion criteria were being an at-risk relative, contacting the clinic by phone, and describing having received a letter. The nurses were instructed to invite both genders and with a broad age range.

One author (A.Ö.) conducted the interviews. An interview guide based on the research questions, existing literature, and the authors’ expertise in clinical cancer genetics, could be referenced as needed to make sure all topics were covered (see supplementary information). The interviews were recorded and transcribed verbatim and lasted 21 to 50 min. The first interview of the study was in person at an office at the university hospital, but because of the pandemic, two of the interviews were conducted via video and eleven via telephone. No authors had any previous or ongoing contact with study participants.

In total, we conducted 14 interviews from September 2020 to April 2022. Before being interviewed, all participants had talked to the genetic counsellor on the phone, some were also waiting for a scheduled appointment, and none had provided a blood sample or received their predictive genetic test results.

One author (A.Ö.) read the transcripts, noted impressions, and coded the text using the software program OpenCode 4.03 [17]. Another author (A.R.) took part in the process on regular occasions, cross-checking transcripts and codes and developing themes. We analysed both manifest and latent codes for similarities and differences and grouped them into themes using thematic maps. We performed the analysis using an inductive thematic approach as presented by Braun and Clark [18–20]. All authors, clinicians with experience in clinical cancer genetics, genetic counselling, and oncology, continually discussed the data and the results.

Although the qualitative results should be seen as a thematic interpretation of the interviews, with no attempt to look for representativity in terms of numbers, we use the terms ‘some’ when 2–7 participants expressed a certain thought or feeling, ‘many’ for 8–11 participants, and ‘most’ for 12–13 participants. We set these cut-offs to facilitate understanding of the data.

### Ethical consideration

This study was approved by the Swedish National Ethical Review Board (application no 2019-02647 and 2020-01176). Participants were given participant research information (see Supplementary information) and signed an informed consent.

## Results

We invited 15 at-risk relatives, one of whom accepted the invitation but did not answer the calls from the first author, and 14 of whom ultimately were interviewed. Participant characteristics are given in Table 1. Below, we present the results of the analysis in two sections. The first section, “Actions and reactions when receiving the letter”, presents a description of the situation that arose when the participants received the letter and their actions and reactions to this situation. The second section, “An important message to hold and to handle for oneself and for others”, presents an overarching theme and related subthemes from the thematic analysis of participants’ experiences and attitudes towards the letter and its message.

**Table 1:** Participants’ characteristics.

Characteristics	*n*
Gender	
Women	9
Men	5
Age (years)	
18–40	5
40–65	4
> 65	5
Area of residence	
Countryside	7
Urban area	4
City	3
Parenthood	
No children	2
Children, underaged or adult	12
Occupational and educational level	
Occupation requiring primary or secondary education or vocational training	8
Occupation requiring tertiary education	6
Associated cancer in a first-degree relative	
Yes	11
No	3
Family diagnosis	
HBOC (variants in *BRCA1/BRCA2/PALB2*)	6
Lynch syndrome (variants in *MLH1/MSH2/MSH6/PMS2*)	7
Familial colorectal cancer (negative genetic screening)	1
Prior knowledge of the familial assessment and of the letter coming	
Yes	9
No	5s

### Actions and reactions when receiving the letter

All participants found it easy to understand the letter’s language and content, and most found the contact information to be complete. All letters contained a family-related serial number, which was perceived as helpful. One letter also contained the patient’s name, at the request of the patient. Participants reported having varying degrees of prior knowledge about familial cancer genetics assessment at the time they received the letter. Many participants had been informed to some degree about the assessment and that a letter was coming. For some participants, the letter arrived without any notice. Two participants had a first-degree relative with a cancer diagnosis associated with a hereditary cancer syndrome. One participant found that the letter made sense as she had been thinking about cancer heredity in the family. Another participant did not have any prior knowledge about cancer in the family and described the letter as evoking many questions about her extended family, risk for cancer, and how the information was presented in the letter. Of those without prior knowledge, most but not all contacted a relative for more information.

After receiving the letter, many participants called the clinic within days or a few weeks, but some waited up to a couple of months. Those who immediately responded to the letter explained their action as arising from wanting to ask the counsellor questions and wanting to take the blood test. Those who waited explained the delay as due to the being occupied with other priorities, such as the Covid-19 pandemic, the birth of a child, or caring for an ill parent. A few said that they had wanted to make contact but just had not gotten around to it, and one participant mentioned feeling guilty towards family. Many expressed a need for genetic counselling before telling their own family members about the letter, and some even wanted to know their own test result first. At the time of being interviewed, about half of the participants had told at least one close relative (a partner, co-parent, sibling, or adult child) about the letter, but not necessarily all family members who could be affected by the information. Some did not have a relative who needed disclosure. Two had not disclosed the information to anyone.

### An important message to hold and to handle for oneself and for others

**Fig. 1:**
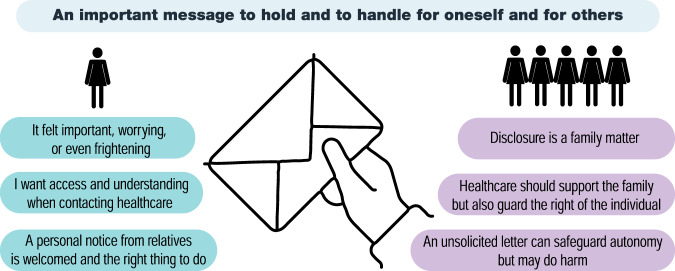
Overview of results. Overarching theme and subthemes reflecting the participants’ experiences and attitudes.

When analysing the interviews regarding the experience of receiving a direct letter and attitudes toward disclosing hereditary cancer risk, we developed an overarching theme and six subthemes (Fig. 1). The overarching theme was “an important message to hold and to handle for oneself and for others”. It summarises that the message of hereditary risk and the letter had to be dealt with. The impact of the letter itself depended on whether and how much the participants were previously informed by their relatives, and being informed helped them cope better. The message evoked mixed feelings and ambivalence around the benefits of accessing risk control and the drawback of knowledge causing worry. They had to process what the message meant, or could mean, for themselves and for their children, co-parents, or grandchildren. All of them were thinking and worrying about how and when to pass on the information to their relatives in turn. They also clearly expressed that disclosing is a family matter but that healthcare should support both patients and relatives when necessary.

One might need to kind of, digest it a little bit … “How should I think about it? What do I think of it? Do I want to know? How do I want to proceed?” (Participant no 5, female, family with Lynch syndrome)

### It felt important, worrying, or even frightening

Those who had been notified about the letter beforehand expressed that the letter was important but concerning. Many wanted the knowledge regardless of if they would take action, and many wanted the possibility to access risk control through control programs. Some had been waiting for the letter. Some described it as an unpleasant reminder of what they already knew or that it made them worried about their and their children’s futures:

When I got the letter, had dad not told me earlier, I wouldn’t have wanted such a letter as it was. (Participant 3, male, family with HBOC)

Even if I knew it [the letter] was coming, it did hurt a little bit when I got it […] it affects you, it does, I’m telling you that. (Participant 4, male, family with HBOC)

The participants who had not known a letter was coming described feeling confused, worried, and even fearful when reading the letter. They described this unsolicited message as difficult to take in and to understand, evoking questions about the family history leading to a genetic assessment, the assessment itself, and the possible physical and psychological impact of risk and risk handling for themselves or their close ones.

I just got the corona vaccine, a kind of ticket [back to normality] and now I get a letter, a new ticket to an insecure future. (Participant 2, female, family with Lynch syndrome)

No one openly questioned the approach of sending letters, but two participants commented on others they knew questioning the appropriateness of the approach.

### I want access and understanding when contacting healthcare

Most of the participants commented on positive experiences with finding concise information in the letter regarding whom and where to call. They expressed that they wanted, expected, or even demanded to have their self-initiated contact call to the clinic answered promptly. When presenting themselves and their case, they wanted the counsellor to be informed and familiar with genetic assessments, the letter, and preferably also their own family. They wanted to have counselling on the phone or to schedule an appointment for counselling and genetic testing. Talking to the counsellor was mostly described as positive and reassuring, however occasionally described as concerning and overwhelming.

I did think she [my mother’s counsellor] would answer but then it wasn’t her, and then I had to answer the kind of questions I had hoped not to. I wished it would’ve been her who knew my mum […] but it went well. (Participant 1, female, family with HBOC)

### A personal notice from relatives is welcomed and the right thing to do

Participants without prior knowledge spontaneously expressed disappointment or anger with relatives who did not notify them that the letter was coming. The participants who had been informed about the assessment and the letter appreciated the prior communication from relatives even if it was brief and from a more distant relation. A general attitude among participants was that it was caring or even correct or decent to personally notify relatives about the familial assessment before healthcare-mediated disclosure.

They could’ve called us and told us that [the index patient/proband] is having a [cancer genetic] assessment and that it means that we also will have to do it. It’s a bit … I feel a little dis … I feel disappointed and slightly angry. For heaven’s sake, we don’t have the best family relations, but when it comes to life or death like this, one thinks that … well … she has to get a hold of herself. (Participant 14, female, family with HBOC)

### Disclosure is a family matter

All participants talked about the responsibility of disclosing to relatives as a family matter without reflecting further. Some expressed their attitude in a more direct way, commenting that making information available to relatives is a duty:

One can draw parallels to Covid and vaccines … the right to decide for yourself what you want to do and what info to take in and how to live, but there must be some responsibility towards others [relatives and people in general] or at least respect for others … one is part of something greater. (Participant 9, male, family with Lynch syndrome)

They considered the act of disclosing to be something that needed to be well thought out and done in a responsible and caring way. Their own plans for disclosure ranged from rehearsing what they would say to waiting for a family gathering to tell people in person to waiting for their own test results to avoid causing relatives worry. Interestingly, no participant remarked on the possibility of asking the cancer genetics clinic to send letters to their relatives in turn.

### Healthcare should support the family but guard the right of the individual

All participants assumed that patients receive support from healthcare professionals around disclosing the results from the genetic assessment. Many thought that patients might need extra support because they might not understand the information or why it is important to disclose. Participants also could imagine the patient not feeling comfortable approaching relatives, not being able to do so because of illness, or not wanting to reveal their own diagnosis. All participants thought healthcare should contact relatives directly if the patient asked for help or if there was a concern that disclosing might not take place:

Healthcare must take responsibility and reach closure even if the patient who started the assessment isn’t coping — healthcare has to support and reach closure. (Participant 7, male, family with HBOC)

… But it’s not that person’s own business anymore if it’s something that can be passed on in the family. (Participant 6, female, family with Lynch syndrome)

Some stated that a patient should not have the right to withhold genetic information from biological relatives. However, one participant mentioned wanting the right to have sufficient time to inform relatives personally before healthcare did. Another participant suggested that knowing a letter was on its way would be a reminder to the patient to notify distant relatives.

### An unsolicited letter can safeguard autonomy but may do harm

The participants thought that healthcare should send a direct letter if necessary to relatives to safeguard their autonomy and agency. These at-risk relatives believed they had the right to know about their risk and that if a counsellor understood that the patient might fail to inform, a letter would be an appropriate measure to take. In general, these participants trusted healthcare and perceived targeted prevention programs as beneficial. Nevertheless, some participants expressed ambivalence about direct contact, the role of healthcare in society, and the effect of targeted prevention programs. There were concerns that direct contact might be seen as offensive or a breach of privacy. In addition, some expressed concern that prevention programs cannot prevent cancer and only offer early detection, and one participant mentioned that such a program introduces a risk of overdiagnosing. Some reflected on the fundamental uncertainty of life and the illusion of having control. Both those with and without prior knowledge of risk when they received the letter commented on the possible negative impact of the unsolicited risk disclosure. If a person who received a letter was in a difficult life situation or had limited resources to handle the message, it could be misinterpreted and do harm.

I feel like a fairly stable person but […] a person who […] maybe has a depression or something, getting a letter like this can be terrible. Also what you don’t know, you cannot control, but you don’t have to worry about it either. (Participant 5, female, family with Lynch syndrome)

One participant commented on an adult sibling needing emotional support from family after being informed and that healthcare has to have a plan for follow-up of those who do not respond to a direct letter.

## Discussion

The practice of family-mediated risk disclosure of hereditary cancer is well established at cancer genetics clinics [21]. As the use of genetic testing broadens, the number of patients increases, and counselling practices change, the limitations in the practice of reaching at-risk relatives should be addressed [22]. In this interview study, we explored experiences and attitudes of at-risk relatives being exposed to such a direct approach of risk disclosure by letter within the context of a randomised clinical trial in a clinical setting.

The study participants found it acceptable to receive a direct letter because they expected it or because they found the information important. They wanted to know no matter what and they wanted access to control programs. The letter and its message often triggered negative feelings such as worry, fear, and confusion. These results are in line with qualitative and questionnaire data from a Danish study of relatives who had received a direct letter disclosing heredity for Lynch syndrome and who found the information emotionally complex but important [14]. Furthermore, 76% of the Danish participants [14] and 91% of participants in a Finnish study   [13] thought it was generally acceptable to be notified about hereditary cancer risk by letter.

The participants in our study who had prior knowledge of the genetic assessment and the incoming letter expressed that these factors helped them cope with the situation. Without prior knowledge, the experience was more complicated as the letter evoked negative feelings and a need for more counselling. After receiving a letter, the participants expected professional handling of their case when contacting the cancer genetics clinic. We think it is a reasonable expectation that if healthcare representatives take the initiative to reach out to relatives directly, they have an increased responsibility regarding access to counselling for those they approached.

A recurrent theme was that participants wanted a prior contact and personal notice from their relatives before healthcare-mediated disclosure; i.e., before receiving a letter, ensuring this personal notification was perceived as the appropriate thing to do. Yet in the Danish study of direct contact, only half of the relatives (49%) thought it was important to be notified about the letter before receiving it [14].

Regarding attitudes towards direct risk disclosure from healthcare, the participants expressed that heredity is a family matter and that relations matter. They reflected on how and when to disclose to their own family, most of them finding the task challenging to some degree. Genetic risk and disclosure are known to be perceived as a familial concern [23–26]. At the same time, the participants approved of a direct approach from healthcare and they believed healthcare should safeguard the rights of relatives. We find it interesting that our participants reflected on how to inform their relatives, and that even though they themselves had received a letter, they did not spontaneously mention the direct approach from healthcare as an option. Some participants expressed concerns about the possibly negative or even harmful aspect of receiving an unsolicited letter from healthcare. According to questionnaire data from the Danish study of direct contact in families with Lynch syndrome, 3% of those receiving a direct letter would have preferred not to have this information at all [14]. Studies quantifying the emotional effect of a direct approach are scarce but point measurements using the State-Trait Anxiety Index in relatives receiving a notification letter indicate that the psychological consequence is similar to that with the family-mediated approach [12, 13]. However, as information on risk may have lifelong implications, it would be interesting to investigate relatives’ long-term understanding and coping.

A strength of this study is that the participants attitudes on direct contact derives from real-life experience in contrast to studies on hypothetical scenarios. The interviews were conducted within 2 weeks of the participant contacting healthcare in an effort to capture the experience of receiving the letter in close proximity in time. However, a possible limitation is that the counsellor answering the call and inviting the participant to an interview also answered questions and gave counselling that could have influenced the participants’ experience of being approached. Another limitation of our study is that we do not cover the experiences of relatives who chose to not contact healthcare, for any reason. In addition, to our knowledge, similar data are not available in previous studies.

Disclosure of genetic information raises ethical issues, mainly concerning autonomy, confidentiality, duty of beneficence, moral responsibility, and feasibility. The rights and duties of patients, relatives, and healthcare professionals are intertwined [27, 28]. We find the current and previous results to be compatible with the suggestion that a direct letter from healthcare can be a complement to the established approach of family-mediated disclosure. This complementarity applies to clinically relevant risks, in these cases a high risk of hereditary cancer for which healthcare offers targeted prevention programs. We suggest that healthcare professionals experienced in genetic counselling can consider disclosing risk by direct letter to at-risk relatives while taking into account the benefits of a family-mediated first contact and that counselling must be easily accessed when relatives want to make contact. Direct contact has to be implemented in a framework of ethical considerations and good practice and tailored for both the individual patient and relatives.

### Supplementary information

Supplementary Information

## Data Availability

Pseudonymised original data analysed during this study are available from the corresponding author on reasonable request.

## Code availability

Code availability is not applicable as this is a qualitative study. Additional data generated or analysed during this study are available on reasonable request.

## References

[1] Sessa C, Balmaña J, Bober SL, Cardoso MJ, Colombo N, Curigliano G (2023). Risk reduction and screening of cancer in hereditary breast-ovarian cancer syndromes: ESMO Clinical Practice Guideline. Ann Oncol.

[2] Seppälä TT, Latchford A, Negoi I, Sampaio Soares A, Jimenez-Rodriguez R, Sánchez-Guillén L (2021). European guidelines from the EHTG and ESCP for Lynch syndrome: an updated third edition of the Mallorca guidelines based on gene and gender. Br J Surg.

[3] van Leerdam ME, Roos VH, van Hooft JE, Balaguer F, Dekker E, Kaminski MF (2019). Endoscopic management of Lynch syndrome and of familial risk of colorectal cancer: European Society of Gastrointestinal Endoscopy (ESGE) Guideline. Endoscopy.

[4] Monahan KJ, Bradshaw N, Dolwani S, Desouza B, Dunlop MG, East JE (2020). Guidelines for the management of hereditary colorectal cancer from the British Society of Gastroenterology (BSG)/Association of Coloproctology of Great Britain and Ireland (ACPGBI)/United Kingdom Cancer Genetics Group (UKCGG). Gut.

[5] National breast cancer care guideline; version: 4.1 [Internet]. Confederation of Regional Cancer Centres in Sweden. 2022. Available from: https://kunskapsbanken.cancercentrum.se/diagnoser/brostcancer/vardprogram/

[6] National colorectal cancer care guideline; version: 3.1 [Internet]. Confederation of Regional Cancer Centres in Sweden. 2023.

[7] Bokkers K, Vlaming M, Engelhardt EG, Zweemer RP, van Oort IM, Kiemeney L (2022). The feasibility of implementing mainstream germline genetic testing in routine cancer care-a systematic review. Cancers.

[8] Grill K, Rosen A (2020). Healthcare professionals’ responsibility for informing relatives at risk of hereditary disease. J Med Ethics.

[9] Ahsan MD, Levi SR, Webster EM, Bergeron H, Lin J, Narayan P (2023). Do people with hereditary cancer syndromes inform their at-risk relatives? A systematic review and meta-analysis. PEC Innov.

[10] Frey MK, Ahsan MD, Bergeron H, Lin J, Li X, Fowlkes RK (2022). Cascade testing for hereditary cancer syndromes: should we move toward direct relative contact? A systematic review and meta-analysis. J Clin Oncol.

[11] Menko FH, van der Velden SL, Griffioen DN, Ait Moha D, Jeanson KN, Hogervorst FBL (2023). Does a proactive procedure lead to a higher uptake of predictive testing in families with a pathogenic BRCA1/BRCA2 variant? A family cancer clinic evaluation. J Genet Couns.

[12] Sermijn E, Delesie L, Deschepper E, Pauwels I, Bonduelle M, Teugels E (2016). The impact of an interventional counselling procedure in families with a BRCA1/2 gene mutation: efficacy and safety. Fam Cancer.

[13] Aktan-Collan K, Haukkala A, Pylvänäinen K, Järvinen HJ, Aaltonen LA, Peltomäki P (2007). Direct contact in inviting high-risk members of hereditary colon cancer families to genetic counselling and DNA testing. J Med Genet.

[14] Petersen HV, Frederiksen BL, Lautrup CK, Lindberg LJ, Ladelund S, Nilbert M (2019). Unsolicited information letters to increase awareness of Lynch syndrome and familial colorectal cancer: reactions and attitudes. Fam Cancer.

[15] Frey MK, Kahn RM, Chapman-Davis E, Tubito F, Pires M, Christos P (2020). Prospective feasibility trial of a novel strategy of facilitated cascade genetic testing using telephone counseling. J Clin Oncol.

[16] Hawranek C, Ehrencrona H, Öfverholm A, Numan Hellquist B, Rosén A. Direct letters to relatives at risk of hereditary cancer – study protocol for a multi-centre randomised controlled trial of healthcare-assisted versus family-mediated risk disclosure at Swedish cancer genetics clinics (DIRECT-study). Trials. 2023;24:810 10.1186/s13063-023-07829-5 PMC10726564 38105176

[17] OpenCode 4.03. Software program. Umeå University, Umeå, Sweden; 2013.

[18] Braun V, Clarke V (2019). Reflecting on reflexive thematic analysis. Qual Res Sport Exerc Health.

[19] Braun V, Clarke V (2020). One size fits all? What counts as quality practice in (reflexive) thematic analysis?. Qual Res Psychol.

[20] Braun V, Clarke V (2006). Using thematic analysis in psychology. Qual Res Psychol.

[21] Mendes Á, Paneque M, Sousa L, Clarke A, Sequeiros J (2016). How communication of genetic information within the family is addressed in genetic counselling: a systematic review of research evidence. Eur J Hum Genet.

[22] Ballard LM, Band R, Lucassen AM (2023). Interventions to support patients with sharing genetic test results with at-risk relatives: a synthesis without meta-analysis (SWiM). Eur J Hum Genet.

[23] Mendes Á, Metcalfe A, Paneque M, Sousa L, Clarke AJ, Sequeiros J (2018). Communication of information about genetic risks: putting families at the center. Fam Process.

[24] Dheensa S, Lucassen A, Fenwick A (2018). Limitations and pitfalls of using family letters to communicate genetic risk: a qualitative study with patients and healthcare professionals. J Genet Couns.

[25] Pedrazzani C, Aceti M, Schweighoffer R, Kaiser-Grolimund A, Bürki N, Chappuis PO (2022). The communication chain of genetic risk: analyses of narrative data exploring proband-provider and proband-family communication in hereditary breast and ovarian cancer. J Pers Med.

[26] Henrikson NB, Blasi P, Figueroa Gray M, Tiffany BT, Scrol A, Ralston JD (2021). Patient and family preferences on health system-led direct contact for cascade screening. J Pers Med.

[27] Dove ES, Chico V, Fay M, Laurie G, Lucassen AM, Postan E (2019). Familial genetic risks: how can we better navigate patient confidentiality and appropriate risk disclosure to relatives?. J Med Ethics.

[28] Kenny J, Burcher S, Kohut K (2020). Ethical issues in genetic testing for inherited cancer predisposition syndromes: the potentially conflicting interests of patients and their relatives. Curr Genet Med Rep.

