# Supplementary material for: The experience of receiving a letter from a cancer genetics clinic about risk for hereditary cancer
Source: Eur J Hum Genet. 2024 Feb 14;32(5):539–44. doi: 10.1038/s41431-024-01551-9 (PMC11061288; doi:10.1038/s41431-024-01551-9)
Supplement: Supplementary file 1 — Supplementary Information [file 41431_2024_1551_MOESM1_ESM.docx]

Supplementary Information

The Supplementary Information consists of a template of the letter, the interview guide and the research participant information.

1. The letter
2. The interview guide
3. Research participant information

A. The letter, here the template for female and male at-risk relatives in the context of BRCA2

You are receiving this letter from a healthcare professional who wants to inform you of a medical investigation that concerns your family/kinship. This information is not urgent. We have sent you this letter by certified mail to ensure the letter is delivered and reaches the right person.

A relative of yours ([name] – *optional to include proband’s name here*) has undergone an investigation at our clinic, and you may thus already be aware of the information below.

In your family, we have identified a pathogenic gene variant that is related to an increased risk of developing certain types of cancers (breast, ovarian, and prostate cancers). It is possible to find out if you have inherited the variant or not by taking a blood sample. You are welcome to contact us for more information on how an investigation is conducted and what this means for yourself and your family.

If it turns out that you do carry the pathogenic gene variant, we can offer you a surveillance programme. The programme for women includes regular exams of breasts and ovaries. Men are offered regular prostate exams. If you carry the variant, we can also offer your adult children information and predictive testing.

If you do not carry the gene variant, you have no increased risk for these types of cancers, and you cannot pass such a variant on to the next generation.

For more information, you are welcome to contact us using the details below. The investigation is offered at a cancer genetics clinic in your region. Details of all cancer genetic clinics in Sweden are available online at www.1177.se/cancergenetik.

Name (Nurse or Physician)
Tel: [telephone number here of the nurse/physician/local clinic]

Webpage on national health platform

Cancer genetic clinic in [insert location here]

1. **The interview guide**

- Can you describe what you thought/felt when you received the notification of a registered letter?
- Can you describe what you thought/felt when you read the letter about hereditary risk of cancer?
- Did you understand the letter? What do you think of the content? Were you looking for more information, contact with relatives?
- How did you receive the very first information about this heredity in your family? Was it through the letter from us or from your relative? Thoughts/feelings on this information?
- Have you previously known or suspected hereditary risk of cancer within your family?
- What do you feel/think now about finding out about heredity risk, for yourself and others/children?
- Have you raised the issue of hereditary risk with your relatives … about your own risk, about the risk of others?
- Thoughts/feelings on the approach of family-mediated and healthcare-mediated direct contact? On the approach of sending a letter? For your family, for others?
- If the relative who receives a message from us that there is heredity in the family would not want to pass on the information, how do you think such a situation should be handled?
- If the healthcare system always chose to inform relatives by letter, what would you think of that? What is good, what is bad? Practical, emotional aspects.
- Is there anything else you would like to comment on? The experience or attitudes, the interview itself?
- What does your everyday life look like?
- Year of birth?
- Professional life/education?
- City/urban/rural living?
- Family situation/children?

1. Research participant information

We are conducting a research study on working methods at the cancer genetics clinics at Regional Cancer Center North, Umeå, and Sahlgrenska Hospital, Gothenburg. In the study, we want to try to understand how you experience knowing that there has been an investigation of hereditary cancer in your family and that there is a confirmed hereditary cancer in the family.

You will receive this letter when you have a relative who has participated in a cancer genetic investigation, and when you have received a registered letter from our clinic with information that this investigation has been done and that the investigation contains information about the risk that could apply to you personally.

Would you be willing to participate in an interview with one of our researchers? The interview takes a maximum of 50 minutes.

The interview aims to increase knowledge about the experience as a healthy person of receiving information about a cancer genetic investigation. We are particularly interested in gaining more knowledge about your thoughts and feelings about how to best share and disseminate information within the family.

To achieve this, we need your help.

If you are interested in participating in an interview, please fill in the answer form on the next page. Write the phone number and email address where we can reach you, and we will soon get back to you to agree on a time.

Participation is, of course, voluntary. As in healthcare, we have a duty of confidentiality. The interview will be recorded, and recorded material will be stored so that unauthorised persons cannot access it. When analysis and reporting are de-identified, no individual person can be identified. All data are handled in accordance with the Personal Data Act (SFS 1998:204). By written application to the Personal Data Controller for the study (Umeå University, 90185 Umeå), you can annually receive information about what data are stored, request correction, and have data deleted from the database.

Feel free to contact us if you have any questions.

Give us or post this reply slip in the enclosed reply mail envelope.
